# Synthesis, Characterization and Solubility Determination of 6-Phenyl-pyridazin-3(2*H*)-one in Different Pharmaceutical Solvents

**DOI:** 10.3390/molecules24183404

**Published:** 2019-09-19

**Authors:** Faiyaz Shakeel, Mohd Imran, Nazrul Haq, Sultan Alshehri, Md. Khalid Anwer

**Affiliations:** 1 Department of Pharmaceutics, College of Pharmacy, King Saud University, P.O. Box 2457, Riyadh 11451, Saudi Arabia; nazrulhaq59@gmail.com (N.H.); salshehri1@ksu.edu.sa (S.A.); 2 Department of Pharmaceutical Chemistry, Faculty of Pharmacy, Northern Border University, P.O. Box 840, Rafha 91911, Saudi Arabia; imran_inderlok@yahoo.co.in; 3 Department of Pharmaceutics, College of Pharmacy, Prince Sattam bin Abdulaziz University, Al-Kharj 11942, Saudi Arabia; mkanwer2002@yahoo.co.in

**Keywords:** 6-phenylpyridazin-3(2*H*)-one, activity coefficient, cardiovascular drug, dissolution thermodynamics, solubility

## Abstract

The current research work proposed the solubility data and solution thermodynamic properties of the cardiovascular agent 6-phenylpyridazin-3(2*H*)-one [PPD] in twelve pharmaceutical solvents at “*T* = 298.2 K to 318.2 K” and “*p* = 0.1 MPa”. The measured solubilities of PPD were regressed well with “van’t Hoff and Apelblat models”. The solid phases of pure and equilibrated PPD were characterized using differential scanning calorimetry and powder X-ray differactometry, and the results suggested no transformation of PPD into solvates/hydrates/polymorphs after equilibrium. The solubilities of PPD in a mole fraction at “*T* = 318.2 K” were noted at a maximum in dimethyl sulfoxide (DMSO, 4.73 × 10^−1^), followed by polyethylene glycol-400 (PEG-400, 4.12 × 10^−1^), Transcutol^®^ (3.46 × 10^−1^), ethyl acetate (EA, 81 × 10^−2^), 2-butanol (2.18 × 10^−2^), 1-butanol (2.11 × 10^−2^), propylene glycol (PG, 1.50 × 10^−2^), isopropyl alcohol (IPA, 1.44 × 10^−2^), ethylene glycol (EG, 1.27 × 10^−2^), ethanol (8.22 × 10^−3^), methanol (5.18 × 10^−3^) and water (1.26 × 10^−5^). Similar tendencies were also noted at other studied temperatures. The results of the “apparent thermodynamic analysis” showed an endothermic and entropy-driven dissolution of PPD in all pharmaceutical solvents. The results of the activity coefficients suggested a maximum interaction at the molecular level in PPD-DMSO, PPD-PEG-400 and PPD-Transcutol, compared with other combination of the solute and solvents.

## 1. Introduction

**Figure 1:**
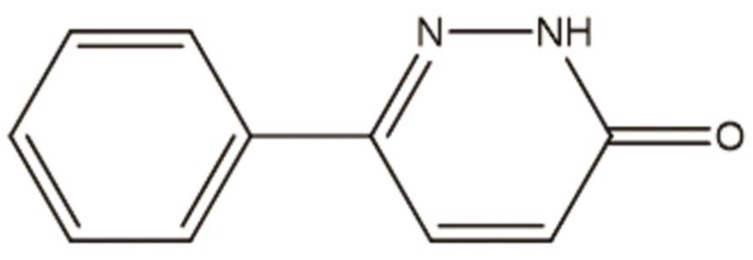
The molecular structure of PPD (molar mass: 172.18 g·mol^−1^).

The derivatives of pyridazinone have been investigated extensively in the control and management of various cardiovascular diseases [1,2]. Some of the pyridazinone derivatives are under various phases of clinical trials while some have been approved for clinical use [3,4,5,6,7]. The compound 6-phenylpyridazin-3(2*H*)-one (PPD) (molecular structure: Figure 1; chemical name: 6-phenylpyridazin-3(2*H*)-one; molecular formula: C_10_H_8_N_2_O; molar mass: 172.18 g·mol^−1^ and CAS registry number: 2166-31-6) occurs as a white crystalline solid [8]. This compound has been studied as a potent cardiotonic agent in the literature [6,8]. Various derivatives of PPD have also been investigated as insecticidal [9], cardioprotective [7,10], analgesics [11,12], anti-inflammatory [12,13], antinociceptive [6], antiulcer [14] and antimicrobial agents [15] in the literature. In spite of various therapeutic activities of PPD derivatives, these compounds have a high toxicity and poor solubility in water and aqueous buffers [1,6]. The poor water solubility of PPD could be a major hurdle for its dosage form design.

The solubility values and other physico-chemical data of newly established compounds and existing ones in various neat pharmaceutical solvents are important in “their synthesis, purification, recrystallization, drug discovery processes and dosage form design” [16,17,18,19]. Hence, it is very important to determine the solubility of PPD in various pharmaceutical solvents in order to get its physicochemical information. In general, the solubility values and solution thermodynamic properties of pyridazinone derivatives have rarely been reported so far. However, the solubilities of a similar compound, 6-phenyl-4,5-dihydropyridazin-3(2*H*)-one, have been recently reported by our research group in eleven different neat solvents including water, ethanol, isopropanol (IPA), ethylene glycol (EG), propylene glycol (PG), polyethylene glycol-400 (PEG-400), 1-butanol, 2-butanol, ethyl acetate (EA), Transcutol and dimethyl sulfoxide (DMSO) at “*T* = 293.2 K to 313.2 K” and “*p* = 0.1 MPa” [20]. The solubilities and solution thermodynamic properties of 6-phenyl-4,5-dihydropyridazin-3(2*H*)-one in different “Transcutol + water” and “PEG-400 + water” binary solvent systems at “*T* = 293.2 K to 313.2 K” and “*p* = 0.1 MPa” have also been reported [21,22]. 

Nevertheless, the solubilities and solution thermodynamic properties of the synthesized compound PPD in any of the investigated pharmaceutical solvents have not been reported so far. Hence, the proposed research work was undertaken to determine the solubilities of PPD (expressed in mole fraction) in twelve different pharmaceutical solvents, including “water, methanol, ethanol, IPA, 1-butanol, 2-butanol, Transcutol, PEG-400, EG, PG, EA and DMSO” at “*T* = 298.2 K to 318.2 K” and “*p* = 0.1 MPa”. The temperature range “*T* = 298.2 K to 318.2 K” was maintained in such a way that the maximum studied temperature, i.e., “*T* = 318.2 K”, should not exceed the melting temperature of PPD (i.e., 474.2 K to 477.2 K) [8]. The maximum studied temperature (i.e., 318.2 K) was lower than the melting temperature of PPD; therefore, this temperature range was maintained in this study. Unlike the temperature, the effect of the pressure on the solubility of PPD was not studied, and hence all these studies were performed at an ambient pressure, i.e., “*p* = 0.1 MPa”. The activity coefficients of PPD in all these pharmaceutical solvents were also determined in order to investigate the interaction of PPD with the respective pharmaceutical solvent at a molecular level. An “apparent thermodynamic analysis” was also conducted for the evaluation of the dissolution behavior of PPD in these pharmaceutical solvents. The results recorded in the proposed research work would be applicable in the “synthesis, recrystallization, purification, pre-formulation studies and dosage form design” of PPD.

## 2. Results and Discussion

### 2.1. Characterization and Identification of PPD

The % yield of PPD was obtained as 77.3 %. The purity of PPD was found to be > 97% (HPLC). The structure elucidation of PPD was confirmed on the basis of “FT-IR spectra, ^1^H-NMR spectra, ^13^C-NMR spectra, mass spectra and elemental analysis”. The recorded FT-IR spectra of PPD are furnished in the Supplementary Figure S1 (Figure S1). The main FT-IR peaks of PPD were noted at 2854.32 cm^−1^, 1529.37 cm^−1^, 1282.13 cm^−1^, 1007.12 cm^−1^ and 587.67 cm^−1^. The ^1^H-NMR spectra of PPD are furnished in Figure S2. The main ^1^H NMR peaks of PPD were noted at different *δ* values. The ^13^C-NMR spectra of PPD are furnished in Figure S3, which also showed the characteristics peaks of PPD at different *δ* values. The mass spectra of PPD are furnished in Figure S4, which suggested the identification of the molar mass of PPD. The resulting data of the elemental analysis of PPD are furnished in Table S1. The mass fractions (%) of C, H and N were found to be 69.75, 4.66 and 16.25 %, respectively, which were very close to the calculated values of C, H and N.

### 2.2. Characterization of Solid Phases of PPD

**Figure 2:**
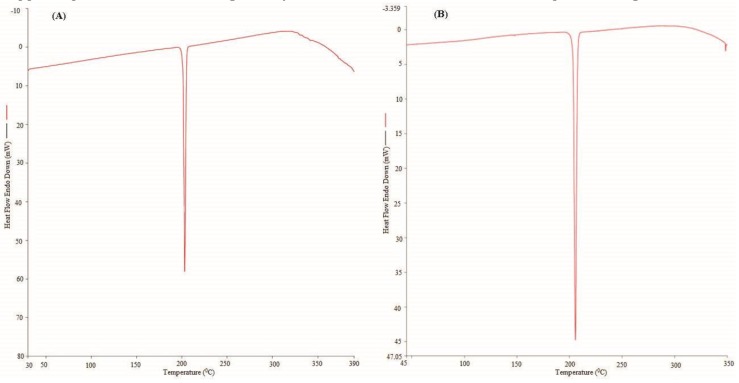
The DSC spectra of (**A**) pure PPD and (**B**) equilibrated PPD; equilibrated PPD was recovered from methanol after slow evaporation.

The characterization of the solid phases of PPD in the pure and equilibrated samples was carried out for the evaluation of the physical form and possible transformation of PPD after equilibrium. “Differential Scanning Calorimetry (DSC) and “Powder X-ray Diffractometry (PXRD)” techniques were applied for this characterization. The recorded DSC spectra of the pure and equilibrated PPD are furnished in Figure 2A,B, respectively. The DSC thermogram of the pure PPD presented a sharp endothermic peak at the fusion temperature (*T*_fus_) = 476.43 K, suggesting the fusion temperature and crystallinity of the pure PPD. The fusion enthalpy (Δ*H*_fus_) of the pure PPD was estimated as 24.51 kJ·mol^−1^ (Figure 2A). The DSC spectra of the equilibrated PPD also presented a sharp endothermic peak at *T*_fus_ = 478.85 K, suggesting the fusion temperature and crystallinity of the equilibrated PPD. The Δ*H*_fus_ = 23.72 kJ·mol^−1^ was recorded for the equilibrated PPD (Figure 2B). There were no significant changes in the DSC profiles of the pure and equilibrated PPD that were noted, which suggested that PPD exists in a pure crystalline form and did not transform into amorphous/polymorphic/hydrates/solvates after equilibrium. The *T*_fus_ value of PPD has been proposed as (474.2 to 477.2) K elsewhere [8]. The *T*_fus_ value of PPD was recorded as 476.43 K in our study. The *T*_fus_ value of the pure PPD recorded in our work was in accordance with the literature value.

**Figure 3:**
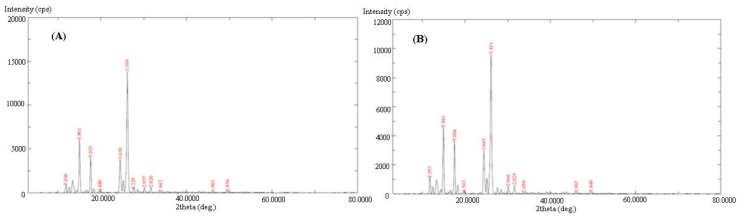
The PXRD spectra of (**A**) pure PPD and (**B**) equilibrated PPD; equilibrated PPD was recovered from methanol after slow evaporation.

The PXRD patterns of the pure and equilibrated PPD are shown in Figure 3A,B, respectively. The PXRD spectra of both samples, i.e., the pure and equilibrated PPD, presented sharp crystalline peaks at various 2 θ values, suggesting the crystalline nature of pure and equilibrated PPD (Figure 3). There were no significant changes in the PXRD patterns of both samples that were noted, suggesting that PPD exists in a pure crystalline form and remained unchanged after equilibrium.

### 2.3. Measured Solubilities of PPD

The measured experimental solubilities of PPD in a mole fraction (*x*_e_) in twelve different pharmaceutical solvents at “*T* = 298.2 K to 318.2 K” and “*p* = 0.1 MPa” are tabulated in Table 1. The solubility values of a similar pyridazinone derivative, i.e., 6-phenyl-4,5-dihydropyridazin-3(2*H*)-one, in eleven different pure solvents, namely “water, ethanol, IPA, EG, PG, PEG-400, 1-butanol, 2-butanol, EA, Transcutol and DMSO” at “*T* = 293.2 K to 313.2 K” and “*p* = 0.1 MPa”, have been reported elsewhere [20]. The solubilities of 6-phenyl-4,5-dihydropyridazin-3(2*H*)-one in various “Transcutol + water” and “PEG-400 + water” binary solvent systems at “*T* = 293.2 K to 313.2 K” and “*p* = 0.1 MPa” were also reported [21,22]. Nevertheless, the solubilities of the investigated compound PPD in any of the neat solvent or binary solvent mixtures have not been reported. 

From the measured solubility data of PPD, it was noted that the *x*_e_ values of PPD were enhanced significantly with respect to the absolute temperature in each pharmaceutical solvent that was studied. The *x*_e_ values of PPD at “*T* = 318.2 K” were obtained at a maximum in DMSO (4.73 × 10^−1^), followed by PEG-400 (4.12 × 10^−1^), Transcutol^®^ (3.46 × 10^−1^), EA (6.81 × 10^−2^), 2-butanol (2.18 × 10^−2^), 1-butanol (2.11 × 10^−2^), PG (1.50 × 10^−2^), IPA (1.44 × 10^−2^), EG (1.27 × 10^−2^), ethanol (8.22 × 10^−3^), methanol (5.18 × 10^−3^) and water (1.26 × 10^−5^). Similar tendencies were also recorded at other temperatures that were studied. The *x*_e_ values of PPD in three pharmaceutical solvents, including “DMSO, PEG-400 and Transcutol”, were much higher when compared with other pharmaceutical solvents that were investigated. Generally, the *x*_e_ values of PPD were recorded as having a similar trend as that reported for the solubility of 6-phenyl-4,5-dihydropyridazin-3(2*H*)-one in eleven different neat solvents. Based on the results recorded in this study, PPD is found to be weakly soluble in water, sparingly soluble in methanol, ethanol, IPA, EG, PG, 1-butanol and 2-butanol, soluble in EA and freely soluble in DMSO, PEG-400 and Transcutol [20,21]. The data recorded in this work could be utilized for the “synthesis, recrystallization, purification, pre-formulation studies and dosage form design” of PPD.

### 2.4. Solubility Parameters for PPD and Various Pharmaceutical Solvents 

The recorded solubility values of PPD were also correlated based on the solubility parameters of the solute and solvent. For this correlation, the “Hansen solubility parameter (*δ*)” for PPD and various pharmaceutical solvents was obtained with the help of Equation (1) [23,24,25]:

(1) $$\delta^{2}=\delta_{d}^{2}+\;\delta_{p}^{2}+\;\delta_{h}^{2},$$

Here, *δ*_d_ = dispersion solubility parameter; *δ*_p_ = polar solubility parameter and *δ*_h_ = hydrogen-bonded solubility parameter. The quantitative values of *δ*, *δ*_d_, *δ*_p_ and *δ*_h_ were obtained using “HSPiP software (version 4.1.07)”. The resulting data is tabulated in Table S2. 

The *δ* value for PPD was calculated as 24.70 MPa^1/2^, suggesting that PPD is a compound with a lower polarity. The solubilities of PPD were also found to be higher in pharmaceutical solvents having lower *δ* values (Table S2). The solubilities of PPD were obtained at their highest in DMSO, which was possibly due to the closed *δ* value of DMSO (23.60 MPa^1/2^) with PPD. Meanwhile, the solubilities of PPD were recorded at their lowest in water, which could possibly be due to the highest *δ* value of water (47.80 MPa^1/2^). Overall, the results of the Hansen solubility parameters were in accordance with the recorded solubility data of PPD.

### 2.5. Theoretical/Ideal Solubilities of PPD 

The theoretical/ideal solubility of pure PPD (*x*^idl^) was calculated using Equation (2) [26]:

(2) $$\ln\;x^{\mathrm{idl}}\;=\frac{-\Delta H_{\mathrm{fus}}\left(T_{\mathrm{fus}}-T\right)}{RT_{\mathrm{fus}}T}+\left(\frac{\Delta C_{p}}{R}\right)[\frac{T_{\mathrm{fus}}-T}{T}+\ln\left(\frac{T}{T_{\mathrm{fus}}}\right)]\;,$$

Here, *R* = universal gas constant and Δ*C*_p_ = difference in the molar heat capacity of the solid state with that of the liquid state [27,28]. 

The Δ*C*_p_ value for PPD was calculated using Equation (3) [26]:

(3) $$\Delta C_{p}=\frac{\Delta H_{\mathrm{fus}}}{T_{\mathrm{fus}}},$$

The quantitative values of *T*_fus_ and Δ*H*_fus_ for pure PPD were obtained as 476.43 K and 24.51 kJ·mol^−1^, respectively, by thermal analysis of pure PPD. The Δ*C*_p_ value for PPD was calculated as 51.44 J·mol^−1^·K^−1^ using Equation (3). With the help of Equation (2), the *x*^idl^ values for PPD were calculated, and the results are tabulated in Table 1. 

The *x*^idl^ values for PPD were recorded in the range of 5.50 × 10^−2^ to 8.22 × 10^−1^ at “*T* = 298.2 K to 318.2 K”. The recorded *x*^idl^ values of PPD were found to be slightly lower than the mole fraction solubilities of PPD in DMSO, PEG-400 and Transcutol at every temperature point. However, these values of PPD were found to be slightly higher than its mole fraction solubilities in EA, 1-butanol and 2-butanol at each of the studied temperatures. On the other hand, the *x*^idl^ values of PPD were found to be much higher than the mole fraction solubilities of PPD in other pharmaceutical solvents such as water, methanol, ethanol, EG, IPA and PG at each of the studied temperatures. Based on these results, DMSO, PEG-400 and Transcutol could be used as the ideal pharmaceutical solvents for the solubility enhancement of PPD. 

### 2.6. Activity Coefficients and Solute-Solvent Molecular Interactions

The quantitative values of the activity coefficients (*γ*_i_) for PPD in twelve different pharmaceutical solvents were calculated using Equation (4) [26,29]:

(4) $$\gamma_{i}=\;\frac{x^{\mathrm{idl}}}{x_{e}},$$

The quantitative values of *γ*_i_ for PPD in twelve different pharmaceutical solvents at “*T* = 298.2 K, 303.2 K, 308.2 K, 313.2 K and 318.2 K” are tabulated in Table 2.

Through the quantitative values of *γ*_i_ for PPD, the solute-solvent interactions at the molecular level can be described. The activity coefficients of PPD were found to be much higher in water compared with other pharmaceutical solvents that were studied. Meanwhile, these values for PPD were noted as being much lower in DMSO, PEG-400 and Transcutol (Table 2) at each investigated temperature. Generally, the values of *γ*_i_ for PPD in most of the pharmaceutical solvents were found to be decreased with a rise in temperature. Based on the estimated values of the activity coefficients, the highest interactions at the molecular level were noted in PPD-DMSO, PPD-PEG-400 and PPD-Transcutol, when compared with other combination of solute and solvent at the molecular level.

### 2.7. Thermodynamic Models for Solubility Correlation 

Various thermodynamic-based mathematical models have been proposed for the correlation of the experimental solubility data of solutes [25,26,27,28,29,30,31]. The most widely applied models for the correlation of the solubility data of solutes in pure/neat solvents are Apelblat and van’t Hoff models [27,28,29,30,31]. Hence, the *x*_e_ values of PPD were correlated with “Apelblat and van’t Hoff models” in the proposed study [25,30,31]. The “Apelblat solubility (*x*^Apl^)” of PPD was estimated using Equation (5) [30,31]:

(5) $$\ln\;x^{\mathrm{Apl}}=\;A+\frac{B}{T}+\;C\ln\left(T\right),$$

Here, *A, B* and *C* = model parameters of the “Apelblat model”. The values of *A, B* and *C* were estimated by applying a “nonlinear multivariate regression analysis” of the *x*_e_ values of PPD, tabulated in Table 1 [22]. The correlation between the *x*_e_ and *x*^Apl^ values of PPD was conducted in terms of “root mean square deviations (*RMSD*) and the coefficient of determination (*R*^2^)”. The *RMSD* values of PPD were estimated by applying its standard equation, as reported in the literature [31,32].

**Figure 4:**
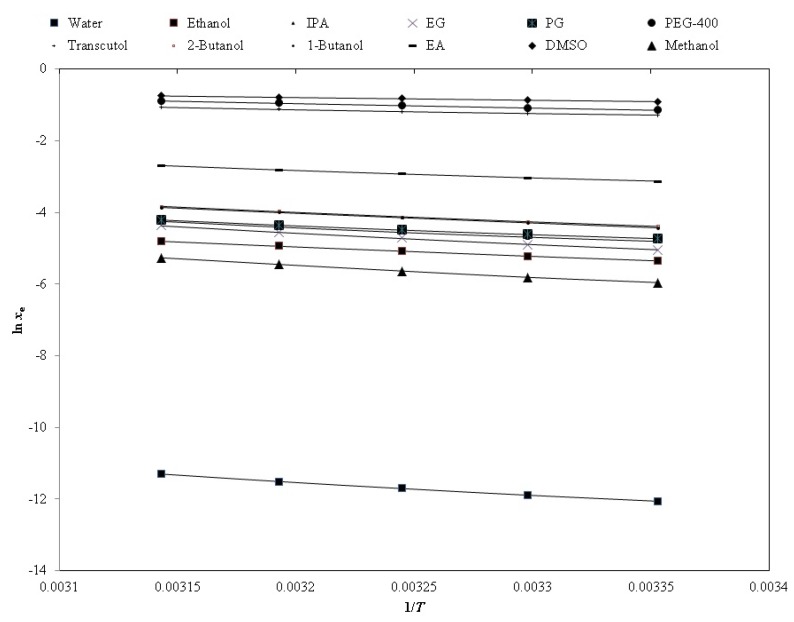
The correlation of the experimental natural logarithmic solubilities (ln *x*_e_) of PPD with the “Apelblat model” in various neat solvents as a function of 1/*T*; the symbols represent the experimental ln *x*_e_ values of PPD, and the solid lines represent the ln *x*^Apl^ values calculated by the “Apelblat model”.

The representative graph for the graphical correlation between the natural logarithmic *x*_e_ (ln *x*_e_) and ln *x*^Apl^ values of PPD in each pharmaceutical solvent against 1/*T* is furnished in Figure 4, suggesting a good correlation between the ln *x*_e_ and ln *x*^Apl^ values of PPD. The estimated values of the “Apelblat correlation” are furnished in Table 3. The *RMSD* values of PPD in twelve different pharmaceutical solvents were obtained as (0.16 to 0.95)%. The overall *RMSD* value for this correlation was recorded as 0.62%. The maximum value of *RMSD* was noted in PG (0.95%). However, the minimum value was noted in DMSO (0.16%). The *R*^2^ values for PPD were noted as 0.9980 to 0.9998. The estimated values of *RMSD* and *R*^2^ suggested a good correlation of the *x*_e_ values of PPD with the “Apelblat model”. 

The “van’t Hoff model solubility (*x*^van’t^)” of PPD was estimated using Equation (6) [25]:

(6) $$\ln\;x^{\mathrm{van}’t}=\;a+\frac{b}{T},$$

Here, *a* and *b* = parameters of “van’t Hoff model”. The values of “*a* and *b*” were estimated from graphs constructed between ln *x_e_* and 1/*T*.

The correlation between the *x*_e_ and *x*^van’t^ values of PPD was conducted again in terms of *RMSD* and *R*^2^. The representative graph for the graphical correlation between the ln *x*_e_ and ln *x*^van’t^ values of PPD in each pharmaceutical solvent against 1/*T* is furnished in Figure S5, suggesting a good correlation between the ln *x*_e_ and ln *x*^van’t^ values of PPD. The estimated values of the “van’t Hoff correlation” are furnished in Table 4. The *RMSD* values of PPD in twelve different pharmaceutical solvents were obtained as (0.70 to 1.70)%. The overall *RMSD* value for this correlation was recorded as 1.16%. The maximum value of *RMSD* was noted in methanol (1.70%) with the minimum one in PEG-400 (0.70%). The *R*^2^ values for PPD were noted as 0.9940 to 0.9991. The estimated values of *RMSD* and *R*^2^ again suggested a good correlation of the *x*_e_ values of PPD with the “van’t Hoff model”. 

### 2.8. Thermodynamic Parameters for PPD Dissolution 

The dissolution behavior of PPD in twelve different pharmaceutical solvents was investigated using an “apparent thermodynamic analysis”. Various thermodynamic parameters, namely “apparent standard enthalpy (Δ_sol_*H*^0^), apparent standard Gibbs free energy (Δ_sol_*G*^0^) and apparent standard entropy (Δ_sol_*S*^0^)”, were estimated via the “apparent thermodynamic analysis”. The “Δ_sol_*H*^0^ values” for the PPD dissolution in each pharmaceutical solvent were estimated at the “mean harmonic temperature (*T*_hm_)” of 308 K by applying “van’t Hoff analysis” using Equation (7) [26,33]:

(7) (∂ln xe∂(1T−1Thm))P= −ΔsolH0R,

The “Δ_sol_H^0^ values” for PPD were obtained by plotting the ln x_e_ values of PPD against 1T−1Thm. The “van’t Hoff plots” in each pharmaceutical solvent were found to be linear with *R*^2^ values of 0.9942 to 0.9992.

The Δ_sol_*G*^0^ and Δ_sol_*S*^0^ values for the PPD dissolution were obtained using Equations (8) and (9), respectively [26,33,34]:

(8) $$\Delta_{\mathrm{sol}}G^{0}=\;-RT_{\mathrm{hm}}\times \mathrm{intercept},$$

(9) $$\Delta_{\mathrm{sol}}S^{0}\;=\;\frac{\Delta_{\mathrm{sol}}H^{0}-\Delta_{\mathrm{sol}}G^{0}}{T_{\mathrm{hm}}},$$

The results of the “apparent thermodynamic analysis” for the PPD dissolution are tabulated in Table 5. 

The “Δ_sol_*H*^0^ values” for the PPD dissolution in twelve different pharmaceutical solvents were estimated as positive values in the range of (6.32 to 30.54) kJ·mol^−1^. The “Δ_sol_*H*^0^ value” for the PPD dissolution was estimated at a maximum in water (30.54 kJ·mol^−1^), followed by methanol (27.51 kJ·mol^−1^), EG (26.88 kJ·mol^−1^), IPA (22.68 kJ·mol^−1^), 1−butanol (22.60 kJ·mol^−1^), 2−butanol (22.42 kJ·mol^−1^), ethanol (21.84 kJ·mol^−1^), PG (20.99 kJ·mol^−1^), EA (17.59 kJ·mol^−1^), PEG-400 (10.31 kJ·mol^−1^), Transcutol (8.93 kJ·mol^−1^) and DMSO (6.32 kJ·mol^−1^). It was noted that the “Δ_sol_*H*^0^ values” for the PPD dissolution were estimated to be lower for pharmaceutical solvents having higher solubility values (DMSO, Transcutol and PEG-400). However, the “Δ_sol_*H*^0^ values” for the PPD dissolution were estimated to be higher for pharmaceutical solvents having lower solubility values (water, methanol and ethanol, etc.). The mean “Δ_sol_*H*^0^ value” for the PPD dissolution was determined as 19.88 kJ·mol^−1^, with a relative uncertainty of 0.38.

The “Δ_sol_*G*^0^ values” for the PPD dissolution in twelve different pharmaceutical solvents were also estimated as positive values in the range of (2.11 to 29.92) kJ·mol^−1^. The “Δ_sol_*G*^0^ value” for the PPD dissolution was also estimated at a maximum in water (29.92 kJ·mol^−1^), followed by methanol (14.39 kJ·mol^−1^), ethanol (12.99 kJ·mol^−1^), EG (12.06 kJ·mol^−1^), IPA (11.61 kJ·mol^−1^), PG (11.46 kJ·mol^−1^), 1−butanol (10.60 kJ·mol^−1^), 2−butanol (10.53 kJ·mol^−1^), EA (7.46 kJ·mol^−1^), Transcutol (3.01 kJ·mol^−1^), PEG-400 (2.59 kJ·mol^−1^) and DMSO (2.11 kJ·mol^−1^). The mean “Δ_sol_*G*^0^ value” for the PPD dissolution was determined as 10.73 kJ·mol^−1^, with a relative uncertainty of 0.68.

The “Δ_sol_*G*^0^ values” of PPD in various pharmaceutical solvents were found to decrease with an increase in the solubility of PPD. The results of the “Δ_sol_*G*^0^ values” for the PPD dissolution were in accordance with the recorded solubility data of PPD. The estimated positive values of “Δ_sol_*H*^0^ and Δ_sol_*G*^0^” in twelve pharmaceutical solvents suggested an “endothermic dissolution” of PPD in all of the studied pharmaceutical solvents [32]. 

The “Δ_sol_*S*^0^ values” for the PPD dissolution in twelve different pharmaceutical solvents were also recorded as positive values in the range of (2.00 to 48.08) J·mol^−1^·K^−1^. The mean value of Δ_sol_*S*^0^ for the PDP dissolution was recorded as 29.71 J·mol^−1^·K^−1^, with a relative uncertainty of 0.43. The recorded positive “Δ_sol_*S*^0^ values” for the PPD dissolution suggested an “entropy-driven dissolution” of PPD in all of the studied pharmaceutical solvents [25]. Finally, the dissolution of PPD was obtained as being “endothermic and entropy-driven” in all of the studied pharmaceutical solvents [25,32]. 

## 3. Materials and Methods

### 3.1. Materials

The compound PPD was synthesized, recrystallized with ethanol, characterized and identified in the “Laboratory of Pharmaceutical Chemistry, Northern Border University, Rafha, Saudi Arabia”. Methanol, ethanol, IPA, 1-butanol and 2-butanol were procured from “Sigma Aldrich (St. Louis, MO, USA)”. Transcutol^®^ was procured as a kind gift sample from “Gattefosse (Lyon, France)”. PEG-400, PG, EA, DMSO and EG were procured from “Fluka Chemica (Buchs, Switzerland)”. Water was acquired from “Milli-Q unit”. The detailed properties of these materials are tabulated in Table S3.

### 3.2. Synthesis of Compound PPD 

For the synthesis of PPD, a mixture of 3-benzoylpropionic acid (0.01 mole) and hydrazine hydrate (0.02 moles) was refluxed in ethanol for about 6 h. The mixture was cooled, and the solid was filtered, washed with water and dried. The solid was then dissolved in acetic acid, and a bromine solution in acetic acid was slowly added to it at *T* = 353.2 K. The resulting mixture was stirred for about 15 min. The obtained solid was filtered, washed with water several times and recrystallized with ethanol in order to obtain the title compound [8]. The scheme for its synthesis is furnished in Figure S6. 

### 3.3. Characterization and Identification of PPD

The synthesized product PPD was characterized for the “yield, purity, FT-IR spectra, ^1^H-NMR spectra, ^13^C-NMR spectra, mass spectra and elemental analyses” [35,36,37]. The standard protocols, as reported in the literature, were used for the characterization and identification of the compound PPD [19,35,36,37].

### 3.4. Quantification of PPD in Solubility Samples

The synthesized compound PPD was quantified in solubility samples by applying a “high performance liquid chromatography (HPLC) connected with ultra-violet (UV)” detector at the wavelength of 254 nm. The entire quantifications were carried out at “*T* = 298.2 K” using “HPLC system (Waters, Milford, MA, USA)”. The column used for this quantification was the “Nucleodur (150 × 4.6mm) RP C_18_ column with 5 μm particle size”. The mixture of methanol and acetic acid (99:1%) was utilized as the mobile phase. The proposed mobile phase was flowed with a flow rate of 1.0 mL·min^−1^ at 254 nm for the elution of PPD. The injection volume was 10 µL for all samples. The standard plot was made between the concentration of PPD and the obtained HPLC area. The standard plot of PPD was noted as linear with a linearity range of (1–100) µg·g^−1^ with an *R*^2^ value of 0.9987. The regressed equation for PPD was recorded as *y* = 82,609*x* + 10,384, where *x* represents the concentration of PPD and *y* represents the obtained HPLC area of PPD.

### 3.5. Characterization of Solid Phases of Pure and Equilibrated PPD

The solid phases of PPD in pure and equilibrated solids were studied using DSC and PXRD. The equilibrated PPD was recovered from methanol by slow evaporation. This characterization was carried out for the evaluation of the physical form and the possible transformation of PPD into polymorphs/solvates/hydrates after equilibrium. The DSC spectra for both samples were obtained using “DSC-8000 Instrument (Perkin Elmer, Milford, MA, USA)”, which was calibrated and equipped with a chiller and autosampler. Accurately weighed amounts of pure PPD (4.80 mg) and equilibrated PPD (5.10) were taken into the DSC pan and sealed hermetically. The temperature range of “*T* = 298.2 K to 673.2 K” was applied for the analysis of both samples. The heating and flow rates were set at 10.0 K·min^−1^ and 20 mL·min^−1^, respectively. 

The PXRD patterns of both samples (pure and equilibrated PPD) were obtained with the help of an “Ultima IV Diffractometer (Rigaku Inc. Tokyo, Japan)”. The 2θ range for obtaining these patterns was set at 3−80°, with a scan speed of 0.5° min^−1^. The rest of the conditions and procedures were the same as those reported for the PXRD analysis of luteolin and thymoquinone in the literature [31,32]. 

### 3.6. Measurement of PPD Solubility in Pharmaceutical Solvents

The solubility of PPD in the investigated pharmaceutical solvents was measured using the equilibrium shake flask method, as reported previously in the literature [38]. The solubility of PPD in each pharmaceutical solvent was estimated at “*T* = 298.2 to 318.2 K” and “*p* = 0.1 MPa”. For these experiments, the excess quantity of PPD was added in known amounts of each pharmaceutical solvent, and each experiment was performed in triplicates. The resultant mixtures were kept for continuous shaking into a “WiseBath^®^ WSB Shaking Water Bath (Model WSB-18/30/-45, Daihan Scientific Co. Ltd., Seoul, Korea)”. The shaking speed and equilibrium time were set at 100 rpm and 3 days, respectively. After 3 days, each sample was taken out from the shaker, and the PPD particles were allowed to settle for about 24 h [22,39]. After settling, the required amounts of supernatants from each sample matrix were withdrawn and diluted with the mobile phase (wherever applicable). The obtained samples were quantified for the PPD contents via the proposed HPLC-UV method at 254 nm. The *x_e_* values of PPD were obtained using Equation (10) [32,39]:

(10) $$x_{e}=\;\frac{m_{1}/M_{1}}{m_{1}/M_{1}+m_{2}/M_{2}},$$

Here, *m*_1_ = mass of PPD (g); *m*_2_ = mass of respective pharmaceutical solvent (g); *M*_1_ = molar mass of PPD (g·mol^−1^); and *M*_2_ = molar mass of respective pharmaceutical solvent (g·mol^−1^). 

## 4. Conclusions

The present studies were undertaken to determine the solubility of PPD in twelve different pharmaceutical solvents at “*T* = 298.2 K to 318.2 K” and “*p* = 0.1 MPa”. The measured solubilities of PPD were recorded as increasing with a rise in temperature in all pharmaceutical solvents. The measured solubilities of PPD regressed well with the “van’t Hoff and Apelblat models”, with overall *RMSD* values of 1.16% and 0.62%, respectively. The solubilities of PPD in the mole fraction at “*T* = 318.2 K” were obtained at a maximum in DMSO, followed by PEG-400, Transcutol, EA, 2-butanol, 1-butanol, PG, IPA, EG, ethanol, methanol and water, and similar tendencies were also recorded at other evaluated temperatures. The values of the activity coefficients that were calculated using ideal solubilities of PPD suggested maximum interactions at the molecular level in PPD-DMSO, PPD-PEG-400 and PPD-Transcutol, compared with other combinations of solute and solvents. The “apparent thermodynamic analysis” showed an “endothermic and entropy-derived dissolution” of PPD in all pharmaceutical solvents that were evaluated. 

## Figures and Tables

**Table 1:** The experimental solubilities (*x*_e_) of PPD in various neat solvents (*S*) at “*T* = 298.2 K to 318.2 K and “*p* = 0.1 MPa” ^a.^

*S*	*x*_e_				
	*T* = 298.2 K	*T* = 303.2 K	*T* = 308.2 K	*T* = 313.2 K	*T* = 318.2 K
Water	5.75 × 10^−6^	6.91 × 10^−6^	8.37 × 10^−6^	1.00 × 10^−5^	1.26 × 10^−5^
Methanol	2.59 × 10^−3^	3.00 × 10^−3^	3.57 × 10^−3^	4.29 × 10^−3^	5.18 × 10^−3^
Ethanol	4.75 × 10^−3^	5.42 × 10^−3^	6.19 × 10^−3^	7.22 × 10^−3^	8.22 × 10^−3^
EG	6.43 × 10^−3^	7.51 × 10^−3^	8.96 × 10^−3^	1.05 × 10^−2^	1.27 × 10^−2^
IPA	8.15 × 10^−3^	9.13 × 10^−3^	1.06 × 10^−2^	1.23 × 10^−2^	1.44 × 10^−2^
PG	8.74 × 10^−3^	9.93 × 10^−3^	1.13 × 10^−2^	1.27 × 10^−2^	1.50 × 10^−2^
1-Butanol	1.19 × 10^−2^	1.36 × 10^−2^	1.59 × 10^−2^	1.84 × 10^−2^	2.11 × 10^−2^
2-Butanol	1.23 × 10^−2^	1.41 × 10^−2^	1.61 × 10^−2^	1.88 × 10^−2^	2.18 × 10^−2^
EA	4.37 × 10^−2^	4.79 × 10^−2^	5.42 × 10^−2^	6.01 × 10^−2^	6.81 × 10^−2^
Transcutol	2.76 × 10^−1^	2.90 × 10^−1^	3.06 × 10^−1^	3.25 × 10^−1^	3.46 × 10^−1^
PEG-400	3.19 × 10^−1^	3.38 × 10^−1^	3.62 × 10^−1^	3.89 × 10^−1^	4.12 × 10^−1^
DMSO	4.03 × 10^−1^	4.19 × 10^−1^	4.38 × 10^−1^	4.55 × 10^−1^	4.73 × 10^−1^
*x* ^idl^	5.50 × 10^−2^	6.10 × 10^−2^	6.75 × 10^−2^	7.45 × 10^−2^	8.22 × 10^−1^

^a^ The standard uncertainties *u* are *u*(*T*) = 0.20 K, *u*(*p*) = 0.003 MPa and *u*_r_(*x*_e_) = 1.61%.

**Table 2:** The values of *γ*_i_ for PPD in various neat solvents (*S*) at “*T* = 298.2 K to 318.2 K” calculated using *x*^idl^ and *x*_e_ values.

*S*	*γ*_i_				
	*T* = 298.2 K	*T* = 303.2 K	*T* = 308.2 K	*T* = 313.2 K	*T* = 318.2 K
Water	9570.00	8840.00	8070.00	7430.00	6550.00
Methanol	21.23	20.30	18.86	17.35	15.87
Ethanol	11.58	11.24	10.90	10.32	10.00
EG	8.55	8.12	7.53	7.08	6.45
IPA	6.75	6.68	6.34	6.06	5.70
PG	6.29	6.14	5.94	5.85	5.47
1-Butanol	4.59	4.46	4.23	4.04	3.89
2-Butanol	4.44	4.30	4.17	3.95	3.77
EA	1.27	1.25	1.24	1.23	1.20
Transcutol	0.19	0.21	0.22	0.22	0.23
PEG-400	0.17	0.18	0.18	0.19	0.19
DMSO	0.13	0.14	0.15	0.16	0.17

**Table 3:** The Apelblat parameters (*A, B* and *C*), *R*^2^ and *RMSD* (%) for PPD in different neat solvents (*S*).

*S*	*A*	*B*	*C*	*R* ^2^	*RMSD* (%)	Overall *RMSD* (%)
Water	−203.37	8364.17	30.54	0.9994	0.91	
Methanol	−521.60	20,859.30	78.22	0.9997	0.74	
Ethanol	−160.38	4887.72	24.33	0.9992	0.57	
EG	−411.70	15,922.29	62.00	0.9998	0.76	
IPA	−494.39	20,155.48	74.06	0.9995	0.87	0.62
PG	−382.75	15,208.33	57.39	0.9986	0.95	
1-Butanol	−131.04	3505.05	20.15	0.9993	0.62	
2-Butanol	−269.06	9860.43	40.65	0.9995	0.42	
EA	−320.51	12,771.75	48.18	0.9995	0.63	
Transcutol	−203.37	8364.17	30.54	0.9994	0.27	
PEG-400	−49.63	1173.90	7.81	0.9980	0.56	
DMSO	−25.60	488.77	4.04	0.9992	0.16	

**Table 4:** The van’t Hoff model parameters (*a* and *b*), *R*^2^ and *RMSD* (%) for PPD in various neat solvents (*S*).

*S*	*a*	*b*	*R* ^2^	*RMSD* (%)	Overall *RMSD* (%)
Water	0.22	−3669.00	0.9970	1.54	
Methanol	5.10	−3304.80	0.9960	1.70	
Ethanol	3.44	−2623.30	0.9985	0.74	
EG	5.76	−3228.70	0.9974	1.44	
IPA	4.30	−2724.80	0.9947	1.69	
PG	3.70	−2521.30	0.9953	1.45	1.16
1-Butanol	4.67	−2715.70	0.9990	0.82	
2-Butanol	4.62	−2693.10	0.9979	1.29	
EA	3.94	−2113.70	0.9961	1.14	
Transcutol	2.30	−1072.50	0.9940	0.75	
PEG-400	3.00	−1238.00	0.9977	0.70	
DMSO	1.63	−759.30	0.9991	0.77	

**Table 5:** Thermodynamic quantities (Δ_sol_*H*^0^, Δ_sol_*G*^0^ and Δ_sol_*S*^0^) and *R*^2^ values for the PPD dissolution in various neat solvents (*S*)^b.^

*S*	Δ_sol_*H*^0^/kJ·mol^−1^	Δ_sol_*G*^0^/kJ·mol^−1^	Δ_sol_*S*^0^/J·mol^−1^·K^−1^	*R* ^2^
Water	30.54	29.92	2.00	0.9971
Methanol	27.51	14.39	42.59	0.9962
Ethanol	21.84	12.99	28.70	0.9986
EG	26.88	12.06	48.08	0.9975
IPA	22.68	11.61	35.93	0.9949
PG	20.99	11.46	30.92	0.9954
1-Butanol	22.60	10.60	38.97	0.9990
2-Butanol	22.42	10.53	38.59	0.9980
EA	17.59	7.46	32.89	0.9962
Transcutol	8.93	3.01	19.20	0.9942
PEG-400	10.31	2.59	25.04	0.9978
DMSO	6.32	2.11	13.64	0.9992

^b^ The relative uncertainties are *u*(Δ_sol_*H*^0^) = 0.38 kJ·mol^−1^, *u*(Δ_sol_*G*^0^) = 0.68 kJ·mol^−1^ and *u*(Δ_sol_*S*^0^) = 0.43 J·mol^−1^·K^−1^.

## Supplementary Materials

The FT-IR, ^1^H NMR, ^13^C NMR and mass spectra of the synthesized compound PPD are presented in Figures S1–S4, respectively. Figure S5 represents the correlation of the experimental solubilities of PPD with the van’t Hoff model. The scheme for the synthesis of the compound PPD is presented in Figure S6. The elemental analysis of the synthesized compound PPD is tabulated in Table S1. The Hansen solubility parameters for the synthesized compound PPD and various pharmaceutical solvents are tabulated in Table S2. The information about materials is furnished in Table S3.
