# Supplementary material for: Synthesis, Characterization and Solubility Determination of 6-Phenyl-pyridazin-3(2H)-one in Different Pharmaceutical Solvents
Source: Molecules. 2019 Sep 19;24(18):3404. doi: 10.3390/molecules24183404 (PMC6767300; doi:10.3390/molecules24183404)
Supplement: Supplementary file 1 [file molecules-24-03404-s001.pdf]

Supplementary materials

Synthesis, Characterization and Solubility Determination of 6-Phenyl-pyridazin-3(2H)-one in Different Pharmaceutical Solvents

Faiyaz Shakeel[*1], Mohd. Imran[2], Nazrul Haq[1], Sultan Alshehri[1] and Md. Khalid Anwer[3]

[1]Department of Pharmaceutics, College of Pharmacy, King Saud University, P.O. Box 2457, Riyadh 11451, Saudi Arabia

[2]Department of Pharmaceutical Chemistry, Faculty of Pharmacy, Northern Border University, P.O. Box 840, Rafha 919111, Saudi Arabia

[3]Department of Pharmaceutics, College of Pharmacy, Prince Sattam bin Abdulaziz University, Al-Kharj, Saudi Arabia

*Author for correspondence

Dr. Faiyaz Shakeel, Associate Professor,

Department of Pharmaceutics,

College of Pharmacy, King Saud University,

P.O. Box 2457, Riyadh 11451, Saudi Arabia

Email: faiyazs@fastmail.fm

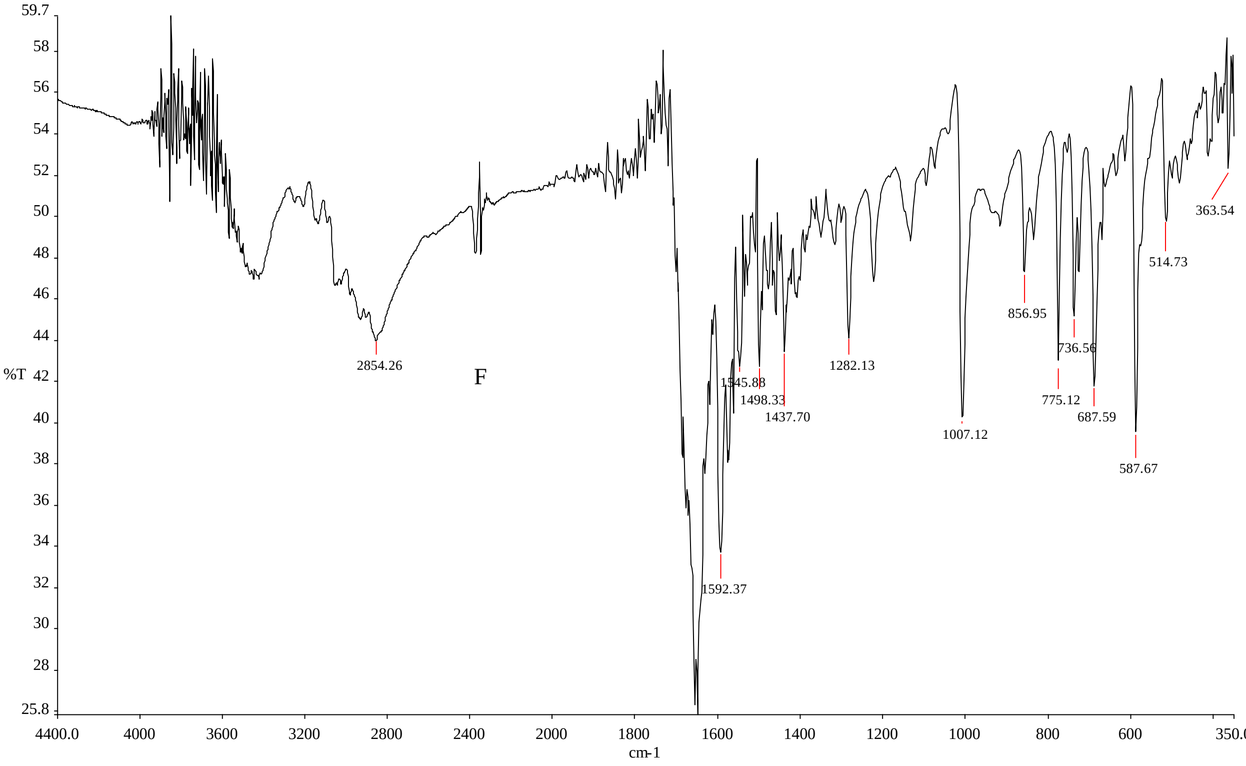

Figure S1. FT-IR spectra of compound PPD showing some characteristic peaks at different wave numbers.

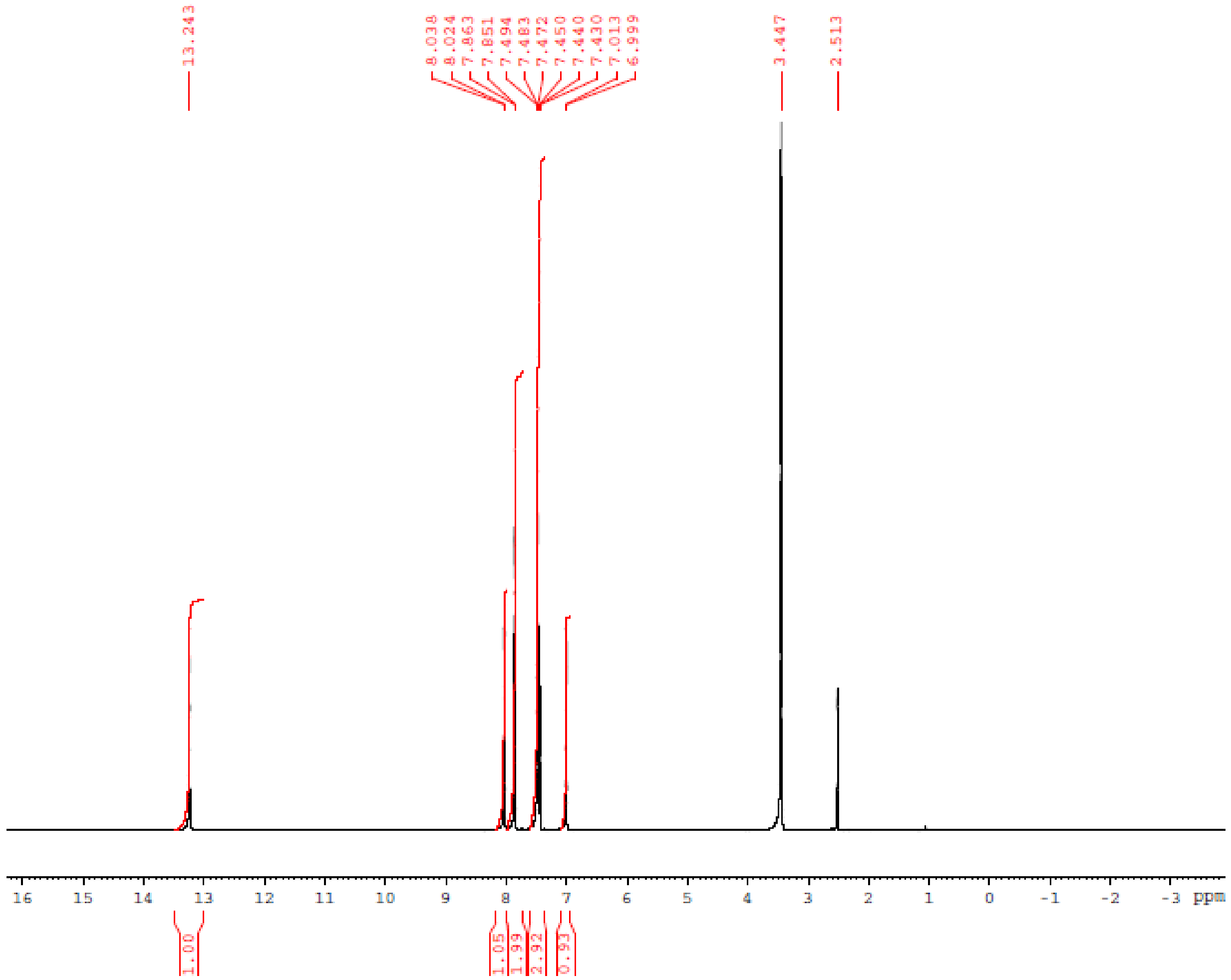

Figure S2: Proton NMR spectra of compound PPD showing some characteristic peaks at different δ values

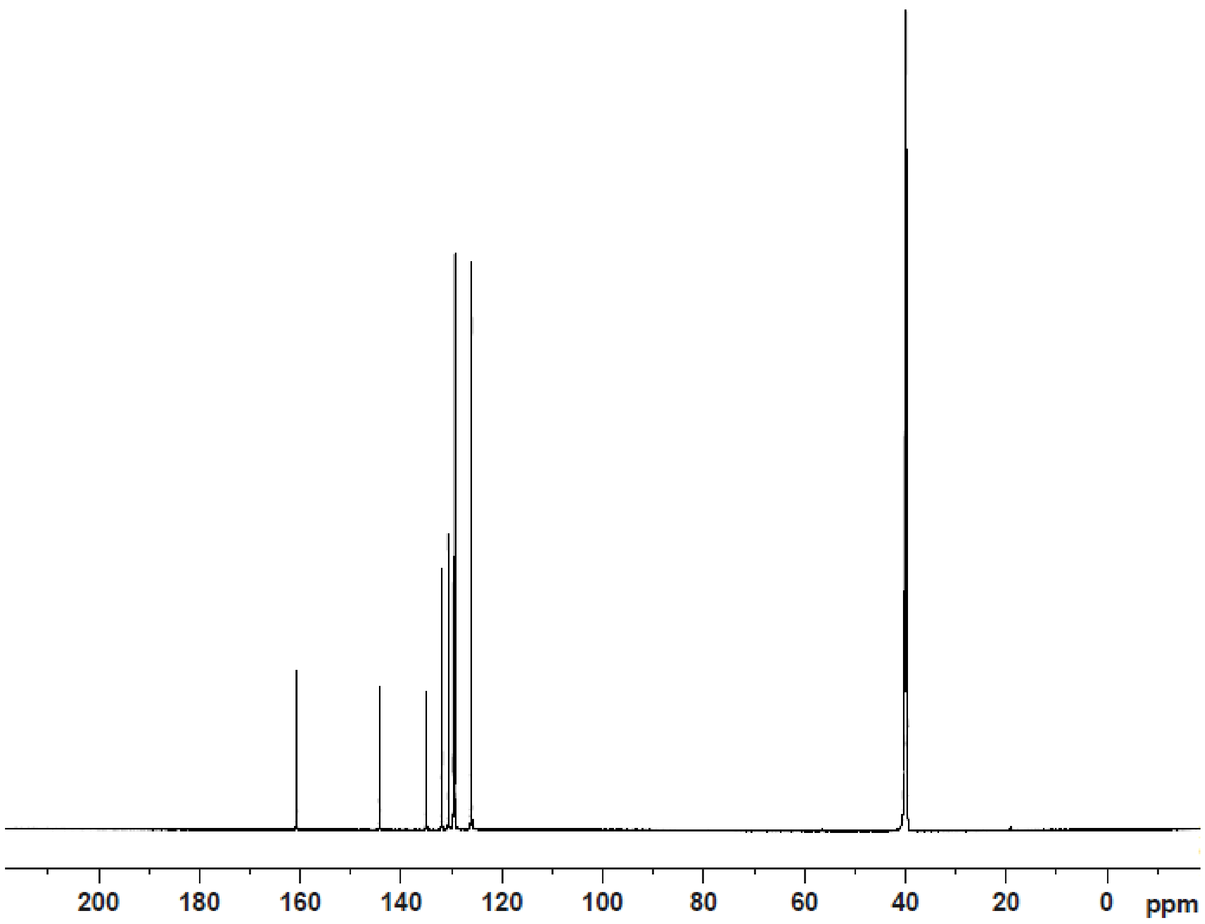

Figure S3: ^{13}C NMR spectra of compound PPD showing some characteristic peaks at different *δ* values

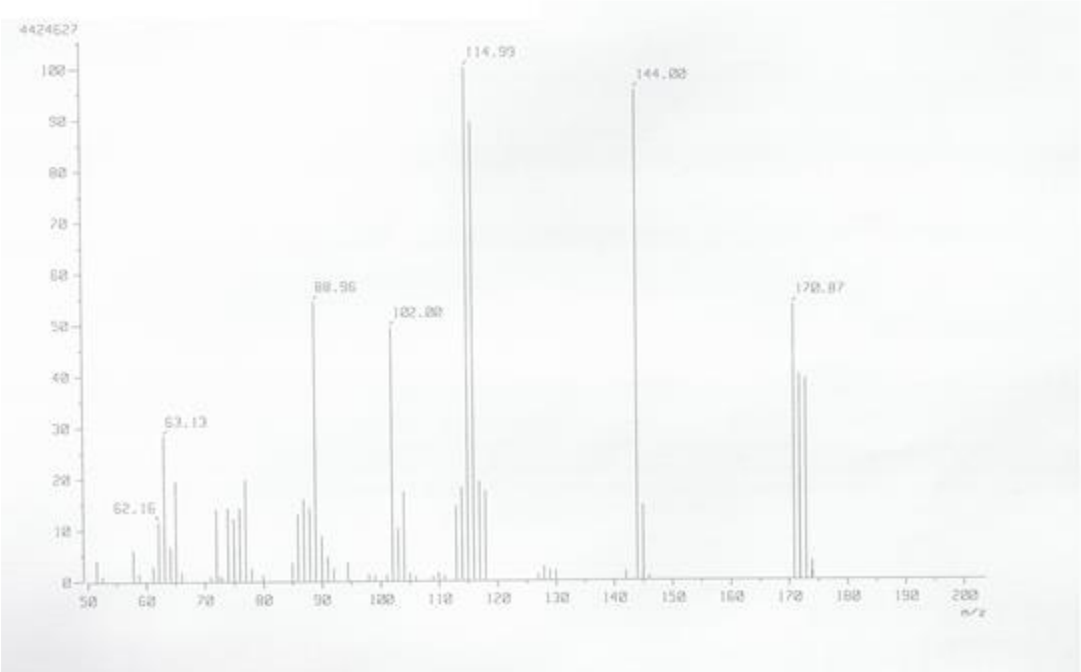

Figure S4: Mass spectra of compound PPD showing some characteristic peaks at different m/z values

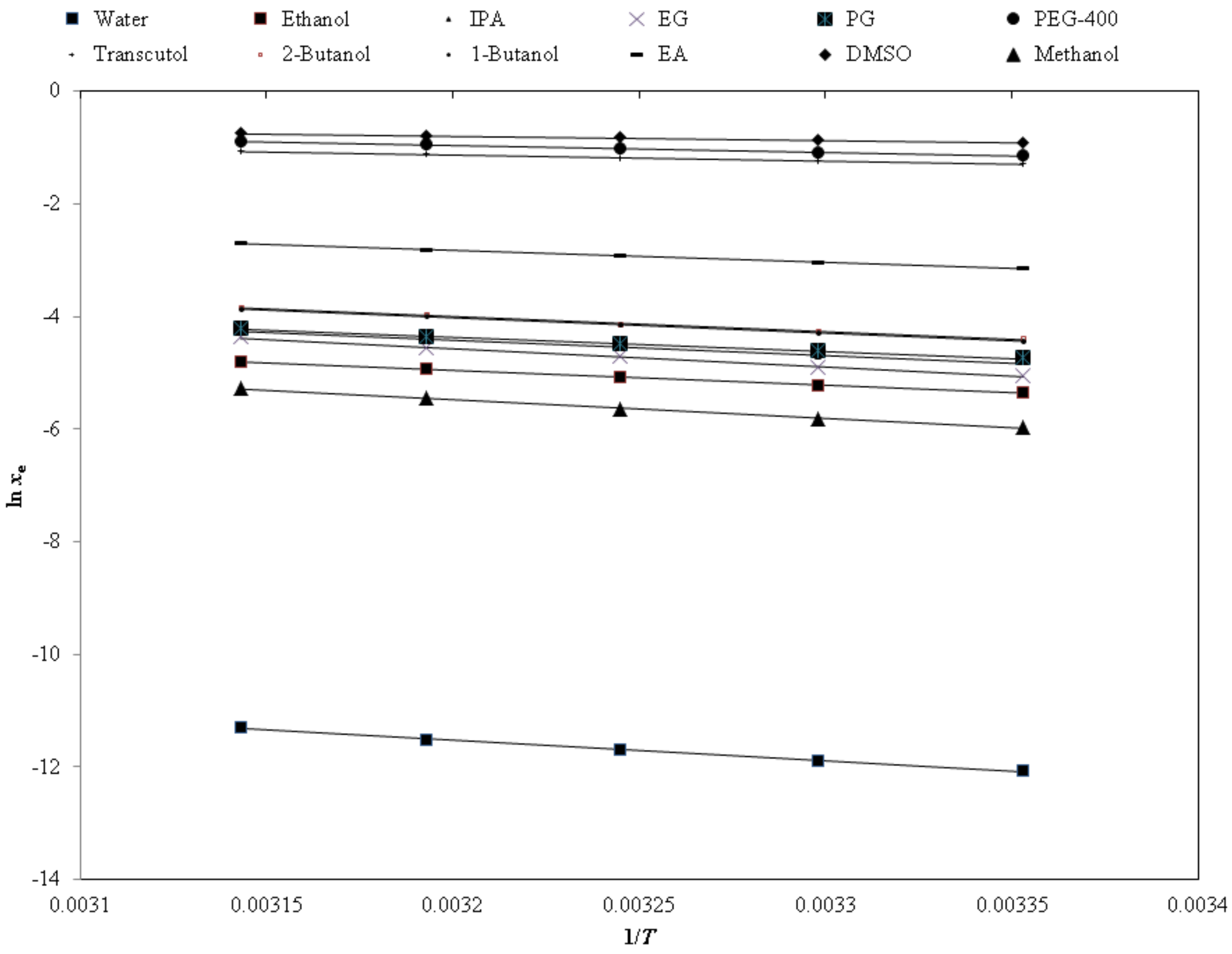

Figure S5: Correlation of experimental natural logarithmic solubilities (ln x_e) of PPD with "van't Hoff model" in various neat solvents as a function of 1/T; symbols represent the experimental ln x_e values of PPD and the solid lines represent the ln $x^{van't}$ values calculated by "van't Hoff model"

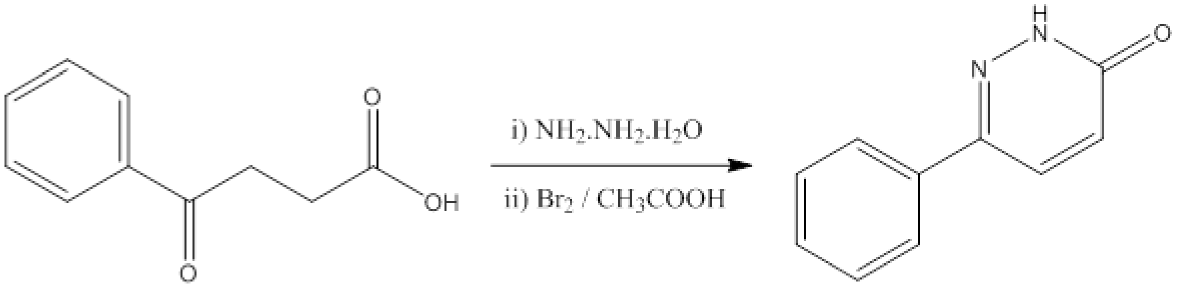

3-Benzoylpropionic acid **PPD**

Figure S6: Scheme for the synthesis of compound PPD

Table S1: Elemental analysis of compound PPD in terms of C, H and N

Compound	Mass fraction of C (%)		Mass fraction of H (%)		Mass fraction of N (%)	
PPD	Calculated	Found	Calculated	Found	Calculated	Found
	69.76	69.75	4.68	4.66	16.27	16.25

Table S2: The δ value of PPD and different neat solvents at T = 298.2 K calculated by HSPiP software

Components	Hansen solubility parameters			
	δ_d/MPa$^{1/2}$	δ_p/MPa$^{1/2}$	δ_h/MPa$^{1/2}$	δ/MPa$^{1/2}$
PPD	20.00	12.90	6.50	24.70
Water	15.50	16.00	42.30	47.80
Ethanol	16.20	8.40	17.60	25.40
Methanol	17.40	10.60	22.40	30.30
PG	17.40	9.10	21.70	29.20
PEG-400	14.60	7.50	9.40	18.90
Transcutol	16.30	7.20	11.90	21.40
EG	18.00	11.10	23.40	31.60
IPA	15.80	6.60	14.30	22.30
1-Butanol	15.90	6.30	15.20	22.90
2-Butanol	15.80	5.40	12.40	20.80
EA	15.70	5.60	7.00	18.10
DMSO	17.40	14.20	7.30	23.60

Table S3: Information about materials used in the experiment

Material	Molecular formula	Molar mass (g mol^{-1})	CAS Registry no.	Purification method	Mass fraction purity	Analysis method	Source
PPD	$C_{10}H_8N_2O$	172.18	2166-31-6	Recrystallization	>0.97	HPLC	Synthesized
Methanol	CH_3OH	32.04	67-56-1	None	>0.99	GC	Sigma Aldrich
Ethanol	C_2H_5OH	46.07	64-17-5	None	>0.99	GC	Sigma Aldrich
EG	$C_2H_6O_2$	62.07	107-21-1	None	>0.99	GC	Fluka Chemica
Transcutol	$C_6H_{14}O_3$	134.17	111-90-0	None	>0.99	GC	Gattefosse
PG	$C_3H_8O_2$	76.09	57-55-6	None	>0.99	GC	Fluka Chemica
PEG-400	$H(OCH_2CH_2)_nOH$	400	25322-68-3	None	>0.99	HPLC	Fluka Chemica
IPA	C_3H_8O	60.10	67-63-0	None	>0.99	GC	Sigma Aldrich
1-Butanol	$C_4H_{10}O$	74.12	71-36-3	None	>0.99	GC	Sigma Aldrich
2-Butanol	$C_4H_{10}O$	74.12	78-92-2	None	>0.99	GC	Sigma Aldrich
DMSO	C_2H_6OS	78.13	67-68-5	None	>0.99	GC	Fluka Chemica
EA	$C_4H_8O_2$	88.11	141-78-6	None	>0.99	GC	Fluka Chemica
Water	H_2O	18.07	7732-18-5	None	-	-	Milli-Q

Both the analysis method and purity were provided by supplier except in case of PPD which was synthesized
